# Lipid based nutrient supplements during pregnancy may improve foetal growth in HIV infected women – A cohort study

**DOI:** 10.1371/journal.pone.0215760

**Published:** 2019-05-02

**Authors:** Minyanga Nkhoma, Per Ashorn, Ulla Ashorn, Kathryn G. Dewey, Austrida Gondwe, Kenneth Maleta

**Affiliations:** 1 Centre for Child Health Research, Faculty of Medicine and Health Technology, Tampere, University, Tampere, Finland; 2 Department of Paediatrics, Tampere University Hospital, Tampere, Finland; 3 Department of Nutrition, University of California Davis, Davis, California, United States of America; 4 School of Public Health and Family Medicine, University of Malawi College of Medicine, Blantyre, Malawi; KU Leuven, BELGIUM

## Abstract

**Objectives:**

Both maternal HIV infection and antiretroviral therapy are associated with adverse birth outcomes. The role of antenatal nutrient supplements with regard to adverse birth outcomes in HIV infected women exposed to antiretroviral therapy is not well known. We assessed the association between HIV and birth outcomes and explored whether antenatal lipid-based nutrient supplements (LNS) modulated this association.

**Methods:**

We analysed a nested cohort of pregnant Malawian women who received daily LNS, multiple micronutrients (MMN) or iron and folic acid (IFA). Birth weight, length-for-age z-score (LAZ) and weight-for-age z-score (WAZ) were analysed as continuous outcomes and proportion of stunting and small-for-gestational age (SGA) as dichotomous outcomes.

**Results:**

134 HIV infected (46 LNS, 39 MMN, 49 IFA) and 833 HIV uninfected (271 LNS, 287 MMN, 275 IFA) women were included. Maternal HIV infection was associated with a lower mean birth weight (-129g (-209, -48), P = 0.002); LAZ (-0.34 (-0.54, -0.13), P = 0.002) and WAZ (-0.21 (-0.40, -0.02), P = 0.041) and a higher risk of stunting (RR (95% confidence interval), 1.87 (1.24, 2.83), P = 0.003) and SGA (1.66 (1.21, 2.26), P = 0.001) in the newborn. If the women received LNS, HIV was not associated with LAZ (mean difference (95%); -0.02 (-0.35, 0.31), P = 0.918) or newborn stunting (RR (95% CI), 0.84 (0.34, 2.03), P = 0.691). However HIV tended to be associated with LAZ if the women received MMN (-0.42 (-0.80, -0.03), P = 0.053); and was significantly associated with LAZ if the women received IFA (-0.52 (-0.89, -0.14), P = 0.021) and with newborn stunting if they received MMN (2.40 (1.15, 4.98), P = 0.029) or IFA (2.40 (1.26, 4.59), P = 0.024).

**Conclusions:**

Further research to investigate the impact of LNS on various aspects of foetal growth in HIV infected women is warranted.

## Introduction

Global estimates indicate that 17.8 million women aged >15 years were living with human immunodeficiency virus (HIV) in 2016 [1]. Following the rapid scale-up of antiretroviral therapy (ART) [2] there has been a considerable reduction in vertical transmission and HIV associated maternal and child mortality [1]. However, maternal HIV continues to be associated with adverse birth outcomes such as preterm birth (PTB) and low birth weight (LBW) even in the context of ART use [3]. Indeed, different aspects of maternal ART including timing of initiation of therapy (pre-conception vs post-conception) or type of antiretroviral regimen have been reported to influence the risk of adverse birth outcomes in HIV infected women [4,5].

Mechanisms for PTB or foetal growth restriction (FGR) in HIV infected women are not well understood. Maternal anaemia, low body-mass-index (BMI), low mid upper arm circumference (MUAC) and low gestational weight gain (GWG) have been identified as risk factors for PTB and FGR in HIV infected women [6–8]. A possible pathway for PTB and FGR is increased oxidative stress levels that have been observed more frequently in HIV infected individuals [9] and in placentas from HIV infected women [10]. Oxidative stress has been linked to PTB and FGR [11,12]. Considering this knowledge, antenatal provision of dietary supplements that may improve maternal nutritional status or reduce oxidative stress may prevent the occurrence of these adverse birth outcomes.

In this study, we evaluated the association between maternal HIV infection and the rate of gestational weight gain (GWG), inadequate GWG and a wide range of birth outcomes (duration of pregnancy, birth weight, length-for age z-score (LAZ), weight-for-age z-score (WAZ), weight-for-length z-score (WLZ), head circumference-for-age z-score (HCZ), PTB, LBW, stunting, underweight, wasting and small-for-gestational age (SGA)). We further examined whether the association between HIV and these pregnancy outcomes would be influenced by lipid-based nutrient supplements (LNS). LNS contain several micronutrients, macrominerals and macronutrients such as protein and essential fatty acids [13]. Alpha linolenic acid, an n-3 fatty acid, has been shown to reduce oxidative stress in rats [14].

## Materials and methods

We conducted a secondary cohort analysis of data collected from pregnant women who participated in the iLiNS-DYAD-Malawi trial (Trial Registration www.clinicaltrials.gov, Identifier: NCT01239693). This was a randomized controlled nutrient intervention trial that evaluated the impact of LNS on pregnancy outcomes. The analysis we are reporting now was defined *a priori* in the iLiNS-DYAD-Malawi study protocol. Ethical approval for the trial was obtained from the College of Medicine Research and Ethics Committee, University of Malawi and the Ethics Committee of Pirkanmaa Hospital District, Finland. Written informed consent was sought from all study participants prior to study participation.

Details of the main iLiNS-DYAD-M trial and the main study results have been described elsewhere [15]. Briefly, we recruited pregnant women who attended antenatal clinics at four health facilities in Mangochi district, a predominantly rural district in South Eastern Malawi. Provision of LNS was not associated with improved birth outcomes [15].

As part of the main trial, participating women received daily oral iron and folic acid (IFA) tablet, multiple micronutrients (MMN) tablet or a sachet of small-quantity LNS throughout pregnancy. The description of the study supplements [15] and the rationale for the formulation of LNS have been reported previously [13]. Briefly, the IFA tablets contained 60 mg of iron (standard dose) whereas the MMN tablets and the LNS sachet contained 20 mg iron each; all 3 supplements contained 400 mcg of folic acid. In addition to IFA the MMN and LNS supplements contained 16 other micronutrients. The LNS also contained 4 macrominerals (phosphorus, calcium, potassium and magnesium) and essential fatty acids (linoleic acid and alpha-linolenic acid) and provided 118 kcal per day.

For this report, we used data that were collected at enrolment (baseline and exposure data) and at two scheduled clinic visits at 32 and 36 gestation weeks (maternal weight), and as soon as possible after birth and at 1–6 weeks postpartum (newborn anthropometry).

The outcome variables of interest were rate of GWG (g/ week), pregnancy duration (gestation weeks), birth weight (grams), LAZ, WAZ, WLZ, HCZ, inadequate GWG (GWG below the lower limit of the recommended weight gain according to the Institute of Medicine’s (IOM) recommendations [16]), PTB (delivery occurring <37 gestation weeks), LBW (birth weight <2500g), stunting (LAZ <-2 at birth) underweight (WAZ<-2 at birth), wasting (WLZ<-2), small head circumference (HCZ <-2 at birth) and SGA (birth weight below the 10th centile of the Intergrowth Standards). Maternal HIV infection at enrolment was the main exposure of interest.

Based on prior axiomatic knowledge, the following baseline variables were defined *a priori* as potential confounders for the relationship between HIV and adverse pregnancy outcomes: maternal age (years), maternal education (number completed years in school), proxy score for socio-economic status (SES) estimated based on household assets adjusted score system [17], BMI (maternal weight (kg) / maternal height (cm)^2^), MUAC (measured in cm to one decimal place), maternal anaemia at enrolment (haemoglobin <110g/l) and maternal *Plasmodium falciparum* infection at enrolment. The type of nutrient supplement received in the iLiNS-DYAD-M trial was considered a potential effect modifier of the relationship between HIV infection and study outcomes.

We determined maternal HIV status on venous blood using the Alere Determine HIV-1/2 Rapid Test (Alere Medical Co) and confirmed positive results using Uni-Gold Rapid Test (Trinity Biotech Co., Wicklow). Use of ART was variable among the HIV infected women who participated in the study. A small proportion of the HIV infected women who already knew their status were using ART prior to conception. Newly diagnosed women or those who were not yet on ART were referred to the health facility’s ART clinic for further management of HIV infection.

We analysed our data using Stata 12.1 (StataCorp, College Station, USA). We restricted our analyses to women who had live singleton births only. Specifically, we excluded women who had no HIV test result, twin gestation, miscarriage, stillbirth, unusual data (gestational age >42 weeks), or incomplete outcome data. The rate of GWG was calculated as the difference between maternal weight measured at the 32/36-wk visit minus maternal weight measured at baseline divided by the number of weeks between these 2 measurements. We defined inadequate GWG as weekly GWG below the lower limit of the Institute of Medicine (IOM) recommended rate for the second and third trimesters (454, 364, 227, 182 g/week for underweight, normal-weight, overweight and obese women respectively) [16].

Since the IOM recommendations are based on pre-pregnancy BMI, we estimated maternal BMI before pregnancy by first determining the best transformation of maternal BMI at enrolment that achieved a normal distribution and then regressed the transformed BMI with age, age squared, and age cubed to generate predicted and residual values. We then inspected the regression curve to determine the earliest gestation age before the confidence interval expanded, with the assumption that weight gain before that point was minimal. Next, we calculated the predicted mean BMI at 13.7 weeks, and this was used as a proxy for pre-pregnancy BMI.

We compared continuous measurements and categorical outcomes between HIV infected and HIV uninfected participants, and between women included or excluded from the analysis. We used linear regression to estimate the coefficient or difference in means. We estimated the risk ratio for the comparison of dichotomous outcomes at a single time point using log binomial regression models or Poisson regression models in case the algorithm failed to converge in the estimation.

To control for possible confounding, we adjusted for covariates that were identified *a priori* as being potentially associated with both HIV infection and FGR. We assessed heterogeneity of effects by stratifying HIV versus No-HIV comparisons by intervention group. We corrected for multiple testing using the false discovery method [18]. Because maternal HIV status was not listed as one of the main potential effect modifiers (hypothesis-driven) in the iLiNS-DYAD-M statistical analysis plan, the stratified analyses were exploratory.

## Results

**Fig 1:**
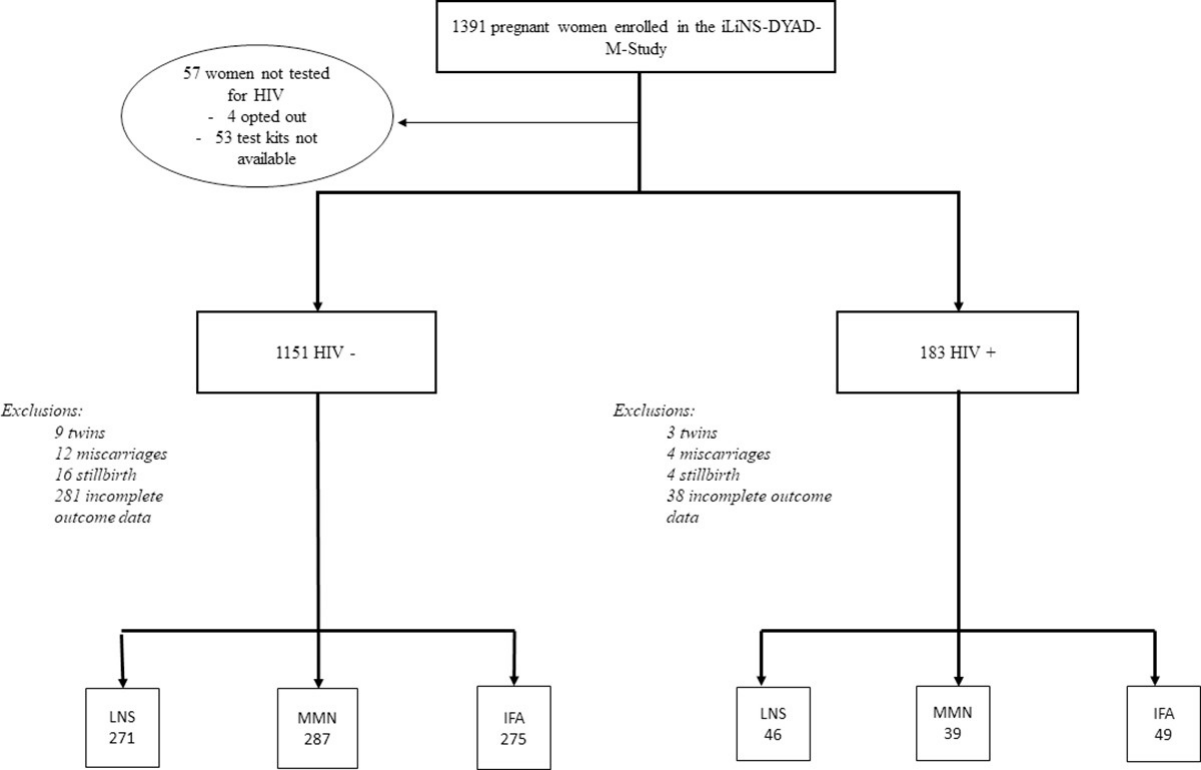
Flow chart of participants included in the analysis.

Out of the 1391 women enrolled into the iLiNS-DYAD-M study, 967 were eligible for analysis. We performed our outcome analyses on this cohort of 134 HIV infected and 833 HIV uninfected pregnant women. Women excluded from the outcome analyses (n = 424) due to missing HIV test results, twin gestation, miscarriage, stillbirth, dropout or incomplete outcome data (Fig 1) tended to be younger (23.9 versus 25.4 y), to be wealthier (0.15 versus -0.05), to be primigravid (31.8% versus 17.5%), to have a higher BMI (22.5 versus 22.0 kg/m^2^) and to be anaemic (24.4% versus 19.0%) but were otherwise similar to women included in the analyses in terms of the other baseline characteristics (S1 Table).

Overall, the rate of attrition was similar between the HIV negative and HIV positive women (27.6 vs 26.8%, P = 0.801). The rates of miscarriage or stillbirth did not differ according to maternal HIV infection (P = 0.259; P = 0.341, respectively). Baseline characteristics of HIV infected, and uninfected women were similar in terms of their height, BMI, education attainment and gestational age at enrolment (P>0.05). However, HIV infected participants tended to be older, to be wealthier, to have a lower haemoglobin level and were less likely to be primigravid (Table 1).

**Table 1:** Baseline characteristics of the women included in the analysis, by HIV status.

Characteristic	No HIV	HIV	P value
Number of participants	833	134	
Mean (SD) maternal age, years	24.9 (6.1)	28.8 (5.2)	**<0.001**
Mean (SD) maternal education, completed years at school	4.0 (3.4)	3.9 (3.6)	0.755
Mean (SD) household assets score	-0.09 (0.92)	0.13 (1.10)	**0.021**
Mean (SD) gestational age at enrolment, weeks	16.8 (2.1)	16.6 (2.2)	0.075
Primigravid, %	19.8	3.0	**<0.001**
Mean (SD) height, cm	156.1 (5.6)	156.2 (5.9)	0.849
Mean (SD) MUAC, cm	26.3 (2.6)	26.6 (2.3)	0.105
Wasting (MUAC<22 cm), %	7.2	4.5	0.246
Mean (SD) BMI, kg/m^2^	22.0 (2.8)	22.3 (2.5)	0.243
Low BMI (< 18.5 kg/m^2^), %	5.7	4.5	0.354
Mean (SD) blood hemoglobin concentration, g/l	112 (16.1)	108 (17.6)	**0.002**
Anaemia (Hb < 100 g/l), %	18.3	23.9	0.125
*P*. *falciparum* infection (RDT), %	22.3	21.6	0.873

MUAC, mid-upper arm circumference; BMI, body-mass-index; RDT, rapid diagnostic testing

P-values were obtained from t-test (comparison of means) or Chi square test (comparison of proportions).

HIV infected women did not differ significantly from HIV uninfected women in terms of pregnancy duration or newborn WLZ or HCZ, but they had a slower rate of GWG (273 vs 297 g/week, P = 0.004) and they delivered babies who were lighter (2882 versus 2987g, P = 0.002) and shorter (LAZ -1.28 versus -0.99, P = 0.002 (Table 2). The proportions of infants with PTB, LBW, stunting and SGA were significantly higher among HIV infected women as compared to HIV uninfected women. However, there were no statistically significant associations between maternal HIV status and proportion of women with inadequate GWG or proportion of infants with wasting, underweight or small head size (Table 3).

**Table 2:** Continuous pregnancy outcomes by HIV infection status^a^.

Outcome	No HIV (N = 833)	HIV (N = 134)	*Unadjusted difference in means (95% CI)*^*b*^	*P-value*^*b*^	*Adjusted difference in means (95% CI)*^*b*^	*P-value*^*b*^
Gestational weight gain rate, g/week	297 (102)	273 (108)	**-25 (-43, -6)**	**0.010**	-28 (-47, -9)	**0.004**
Duration of pregnancy, weeks	39.4 (1.60)	39.3 (1.84)	-0.11 (-0.41, 0.19)	0.470	-0.11 (-0.42, 0.20)	0.493
Birth weight, g	2987 (425)	2882 (447)	-105 (-183, -27)	**0.009**	-129 (-209, -48)	**0.002**
Length-for-age z-score	-0.99 (1.10)	-1.27 (1.16)	-0.29 (-0.49, -0.08)	**0.006**	-0.34 (-0.54, -0.13)	**0.002**
Weight-for-age z-score	-0.55 (1.01)	-0.69 (1.07)	-0.14 (-0.33, 0.04)	0.135	-0.21 (-0.40, -0.02)	**0.041**
Weight-for-length z-score	0.10 (1.13)	0.21 (1.07)	0.10 (-0.11, 0.31)	0.330	0.05 (-0.17, 0.26)	0.663
Head circumference z-score	-0.15 (1.08)	-0.25 (1.20)	-0.10 (-0.30, 0.10)	0.337	-0.13 (-0.34, 0.08)	0.223

^a^ Values are means (standard deviations) unless otherwise indicated

^b^Differences in means and P-values were estimated using linear regression models

Models were adjusted for the following maternal factors: age, education, proxy score for socio-economic status (SES), body-mass-index, mid-upper arm circumference, anaemia at enrolment and *Plasmodium falciparum* infection at enrolment.

**Table 3:** Adverse pregnancy outcomes by HIV infection status^a^.

Outcomes	No HIV (N = 833)	HIV (N = 134)	Unadjusted Risk ratio (95% CI)^b^	P-value^b^	Adjusted Risk Ratio (95% CI) ^b^	P-value^b^
Inadequate gestational weight gain^c^	597 (72.2)	99 (73.9)	1.02 (0.83, 1.27)	0.831	1.06 (0.85. 1.33)	0.594
Preterm birth (<37 gestation weeks)	47 (5.6)	16 (11.9)	**2.12 (1.24, 3.62)**	**0.006**	**1.99 (1.14, 3.46)**	**0.015**
Low birth weight (<2500g)	98 (11.8)	26 (19.4)	**1.65 (1.11, 2.44)**	**0.012**	**1.81 (1.20, 2.73)**	**0.005**
Newborn stunting (LAZ<-2)	124 (14.9)	33 (24.6)	**1.65 (1.18, 2.32)**	**0.003**	**1.87 (1.24, 2.83)**	**0.003**
Newborn underweight (WAZ<2)	56 (6.7)	13 (9.7)	1.44 (0.81, 2.56)	0.211	1.56 (0.86, 2.83)	0.144
Newborn wasting (WLZ<-2)	31 (3.7)	2 (1.5)	0.40 (0.10, 1.66)	0.207	0.45 (0.10, 1.93)	0.282
Newborn small head size (HCZ<-2)	31 (3.7)	9 (6.7)	1.80 (0.88, 3.71)	0.108	1.81 (0.86, 3.80)	0.119
Small-for-gestational-age (birth weight below 10^th^ percentile of birth weight for age (Intergrowth Standards)	235 (28.2)	55 (41.0)	**1.45 (1.16, 1.83)**	**0.001**	**1.66 (1.21, 2.26)**	**0.001**

^a^Values are n (%) unless otherwise indicated

^b^Risk ratio and P-values were estimated using log binomial models or Poisson regression models

^c^Below the lower cutoff of the Institute of Medicine’s recommended range [16]

All models were adjusted for age, education, proxy score for socio-economic status (SES), body-mass-index, mid-upper arm circumference, anaemia at enrolment and *Plasmodium falciparum* infection at enrolment.

The numbers randomised to receive LNS, MMN and IFA were 46, 39 and 49, and 271, 287 and 275 in the HIV infected and HIV uninfected women respectively. Stratified analyses for continuous birth outcomes that were significantly associated with HIV infection in the main analysis showed that the values for birth weight, LAZ and WAZ for HIV infected women who received LNS were consistently closer to those of uninfected women regardless of the type of the intervention received by the uninfected women. Comparisons between HIV infected and uninfected women indicated that for all outcomes the values were mostly lower among infected versus non-infected women in each of the intervention groups, though these differences were statistically significant only for birth weight in the MMN group and LAZ in the IFA group (Table 4).

**Table 4:** Continuous pregnancy outcomes by HIV status, stratified by intervention group^a^.

Outcome	No HIV Mean (95% CI)	HIV Mean (95% CI)	Adjusted difference in means (95% CI)^b^	P-value^b^	PFDR^c^
Gestational weight gain rate, (g/week)					
LNS	298 (105)	278 (105)	-20 (-53, 13)	0.231	0.231
MMN	298 (101)	263 (113)	-42 (-76, -7)	0.017	0.051
IFA	297 (101)	276 (107)	-29 (-62, 4)	0.082	0.123
Mean (SD) birth weight, g					
LNS	2996 (420)	2988 (478)	-143 (-125, 153)	0.841	0.841
MMN	3004 (426)	2822 (457)	-192 (-339, -45	0.011	**0.033**
IFA	2962 (430)	2826 (394)	-151 (-289, -12)	0.033	0.051
Mean (SD) length-for-age z-score					
LNS	-0.95 (1.04)	-0.98 (0.91)	-0.02 (-0.35, 0.31)	0.918	0.918
MMN	-0.96 (1.08)	-1.38 (1.33)	-0.42 (-0.80, to -0.03)	0.035	0.053
IFA	-1.05 (1.16)	-1.47 (1.19)	-0.52 (-0.89, -0.14)	0.007	**0.021**
Mean (SD) weight-for-age z-score					
LNS	-0.51 (0.96)	-0.37 (0.95)	0.10 (-0.21, 0.41)	0.516	0.516
MMN	-0.53 (1.01)	-0.92 (1.10))	-0.40 (-0.76, -0.05)	0.027	0.081
IFA	-0.60 (1.05)	-0.83 (1.08)	-0.31 (-0.65, 0.023)	0. 067	0.101

LNS, lipid-based nutrient supplements; MMN, multiple micronutrient supplements; IFA, iron folic acid; and PFDR, positive false discovery rate. Women with no HIV: LNS (N = 271); MMN (N = 287) and IFA (N = 275). Women with HIV: LNS(N = 46), MMN (39), IFA (49).

^a^ Values are means (standard deviations) unless otherwise indicated

^b^Differences in means and P-values were estimated using linear regression models

^c^P value adjusted for multiple testing using false discovery rate method [18]

All models were adjusted for age, education, proxy score for socio-economic status (SES), body-mass-index, mid-upper arm circumference, anaemia at enrolment and *Plasmodium falciparum* infection at enrolment

Like the findings from the stratified analyses of continuous outcomes, the risk of preterm birth, and stunting in HIV infected women who received LNS was closer to the risk observed in HIV uninfected women regardless of type of supplement received by the uninfected women. Maternal HIV was not associated with LBW or SGA in any of the three intervention groups. In contrast, the risk of PTB was significantly higher among infected versus uninfected women only among those in the MMN group and not in the IFA or LNS groups. The risk of newborn stunting was significantly higher among infected versus uninfected women in the IFA and MMN groups but similar between infected and uninfected women who received LNS (Table 5).

**Table 5:** Adjusted dichotomous pregnancy outcomes by HIV status, stratified by intervention group^a^.

Outcome	No HIV N (%)	HIV N (%)	Risk Ratio (95% CI)^b^	P-value^b^	PFDR^c^
Preterm birth (<37 gestation weeks)					
*LNS*	13 (4.8)	2 (4.4)	0.96 (0.20, 4.51)	0.958	0.958
*MMN*	15 (5.2)	7 (17.5)	4.79 (1.84, 12.5)	0.001	**0.003**
*IFA*	19 (6.9)	7 (14.3)	1.83 (0.68, 4.94)	0.235	0.235
Low birth weight (<2500g)					
*LNS*	31 (11.4)	8 (17.4)	1.74 (0.75, 4.03)	0.193	0.243
*MMN*	34 (11.9)	7 (17.5)	1.66 (0.71, 3.87)	0.243	0.243
*IFA*	33 (12.0)	11 (22.5)	1.94 (0.91, 4.96)	0.088	0.243
Newborn stunting (LAZ<-2)					
*LNS*	43 (15.9)	6 (13.0)	0.84 (0.34, 2.03)	0.691	0.691
*MMN*	38 (13.2)	11 (27.5)	2.40 (1.15, 4.98)	0.019	**0.029**
*IFA*	43 (15.6)	16 (32.7)	2.40 (1.26, 4.59)	0.008	**0.024**
Small-for-gestational-age (birth weight below 10th percentile of birth weight for age (Intergrowth Standards)					
LNS	81 (29.9)	15 (32.6)	1.13 (0.64, 2.02)	0.688	0.688
MMN	75 (26.1)	16 (40.0)	1.65 (0.91, 2.97)	0.098	0.147
IFA	79 (28.7)	25 (51.0)	2.12 (1.29, 3.47)	0.003	0.099

LNS, lipid-based nutrient supplements; MMN, multiple micronutrient supplements; IFA; iron folic acid; and PFDR, positive false discovery rate. Women with no HIV: LNS (N = 271); MMN (N = 287) and IFA (N = 275). Women with HIV: LNS(N = 46), MMN (39), IFA (49).

^a^Values are n (%) unless otherwise indicated

^b^Risk ratio and P-values were estimated using log binomial models or Poisson regression models

^c^P value adjusted for multiple testing using false discovery rate method [18]

All models were adjusted for age, education, proxy score for socio-economic status (SES), body-mass-index, mid-upper arm circumference, anaemia at enrolment and *Plasmodium falciparum* infection at enrolment

Out of the 134 HIV infected participants, 38 (28.4%) received ART during pregnancy (20 started ART prior to joining the study and 18 after enrolment into the study) and 10 (7.5%) started ART after delivery; for 30 (22.3%) women it was unknown when they started therapy and for 43 (32.1%) treatment status was unknown. ART status was not associated with type of nutrient supplement received in the main trial (P = 0.702). Because ART use during pregnancy may increase the risk of adverse birth outcomes [19], we conducted a sensitivity analysis in which we excluded all HIV positive women who were on ART (Tables 6 and 7).

**Table 6:** Sensitivity analysis: HIV and continuous pregnancy outcomes after excluding HIV positive women who were on antiretroviral therapy^a^.

Outcome	No HIV (N = 833)	HIV (N = 134)	*Unadjusted difference in means (95% CI)*^*b*^	*P-value*^*b*^	*Adjusted difference in means (95% CI)*^*b*^	*P-value*^*b*^
Gestational weight gain rate, g/week	297 (102)	263 (101)	**-34 (-56, -13)**	**0.002**	**-36 (-57, -14)**	**0.001**
Duration of pregnancy, weeks	39.4 (1.60)	39.1 (1.76)	-0.33 (-0.67, 0.01)	0.061	-0.34 (-0.69, 0.00)	0.052
Birth weight, g	2987 (425)	2890 (446)	**-98 (-188, -7)**	**0.034**	**-123 (-213, -32)**	**0.008**
Length-for-age z-score	-0.99 (1.10)	-1.25 (1.092)	**-0.27 (-0.50, 0.04)**	**0.024**	**-0.33 (-0.56, -0.10)**	**0.005**
Weight-for-age z-score	-0.55 (1.01)	-0.71 (1.02)	-0.16 (-0.38, 0.05)	0.131	**-0.27 (-0.51, -0.02)**	**0.036**
Weight-for-length z-score	0.10 (1.13)	0.12 (1.07)	0.02 (-0.22, 0.26)	0.868	-0.01 (-0.25, 0.24)	0.957
Head circumference z-score	-0.15 (1.08)	-0.30 (1.11)	-0.15 (-0.37, 0.08)	0.213	-0.17 (-0.41, 0.06)	0.143

^a^ Values are means (standard deviations) unless otherwise indicated

^b^Differences in means and P-values were estimated using linear regression models

**Table 7:** Sensitivity analysis: Adjusted adverse pregnancy outcomes by HIV infection status after excluding HIV positive women who were on antiretroviral therapy^a^.

Outcomes	No HIV (N = 833)	HIV (N = 134)	Unadjusted Risk ratio^b^ (95% CI)	P-value^b^	Risk Ratio (95% CI) ^b^	P-value^b^
Inadequate gestational weight gain^c^	597 (72.2)	74 (77.1)	1.07 (0.84, 1.36)	0.595	1.11 (0.87, 1.42)	0.399
Preterm birth (<37 gestation weeks)	47 (5.6)	1313.58)	**2.40 (1.35, 4.27)**	**0.003**	**2.34 (1.30, 4.19**	**0.004**
Low birth weight (<2500g)	98 (11.8)	17 (17.7)	1.50 (0.94, 2.41)	0.088	**1.62 (1.00, 2.63)**	**0.049**
Newborn stunting (LAZ<-2)	124 (14.9)	24 (25.0)	**1.68 (1.15, 2.46)**	**0.008**	**1.93 (1.23, 3.05**	**0.004**
Newborn underweight (WAZ<2)	56 (6.7)	8 (8.3)	1.24(0.61 2.52)	0.553	1.31 (0.63, 2.71)	0.465
Newborn wasting (WLZ<-2)	31 (3.7)	2 (2.08)	0.56 (0.14, 2.30)	0.421	0.56 (0.13, 2.40)	0.438
Newborn small head size (HCZ<-2)	31 (3.7)	5 (5.2)	1.40 (0.56, 3.51)	0.474	1.37 (0.53, 3.50)	0.514
Small-for-gestational-age (birth weight below 10^th^ percentile of birth weight for age (Intergrowth Standards)	235 (28.2)	39 (40.6)	**1.44 (1.10, 1.88)**	**0.007**	**1.62 (1.14, 1.30)**	**0.007**

^a^Values are n (%) unless otherwise indicated

^b^Risk ratio and P-values were estimated using log binomial models or Poisson regression models

The associations between maternal HIV and GWG, birth weight, LAZ, WAZ, preterm birth, LBW, newborn stunting and SGA persisted (P<0.05) after excluding those who were on ART. Notably, the associations between HIV and LBW, newborn stunting and SGA were attenuated after excluding those who were on ART whereas the association between maternal HIV and PTB strengthened. We did not collect information on the exact ARV drugs received by the individual HIV positive women participating in the study. However, in general the ARV regimen for pregnant women in Malawi at the time of the study included stavudine, lamivudine, nevirapine, zidovudine, efavirenz, tenofovir and lopinavir/ritonavir. Since private ART care was rare in the area it is likely that most of the women were on this regimen. There was one case of spina bifida identified in an infant born to an HIV positive woman.

Because previous studies conducted in the pre-ART era showed that HIV was associated with a smaller head circumference [20], we explored whether the mean head circumference differed according to maternal ART use in our study. After adjusting for possible confounders, our results showed that there was no difference in the mean head circumference of infants born to HIV positive mothers who were receiving ART when compared to ART naïve HIV positive mothers (Mean difference HCZ (95% CI), 0.23 (-0.23, 0.70); P = 0.332).

Since there was a considerable loss to follow up, we performed an intention to treat analysis where all individuals excluded from the analysis because of miscarriages, stillbirth or incomplete/improbable data were assumed to have had a poor outcome. Those with twin gestation or missing HIV results were not included in this analysis because twin gestation itself is a risk factor for adverse outcomes and HIV status was required as the independent variable. Out of the 1322 participants included in the sensitivity analysis, 645 infants (including 336 who were defined as being SGA) were considered to have FGR. Maternal HIV infection was associated with FGR (1.27 (1.01, 1.59, P = 0.039).

## Discussion

We investigated the association between maternal HIV and pregnancy outcomes, and whether this association differed across nutrient intervention groups in a cohort of women participating in a nutrient supplementation trial in southern rural Malawi. The study findings show that maternal HIV status was inversely associated with weekly GWG, birth weight, LAZ, and WAZ and positively associated with PTB, LBW, stunting and being born SGA. HIV infected women who received LNS tended to have values of birth size or risk of adverse outcomes similar to that of HIV uninfected women regardless of the type of supplement received by the uninfected women. The association between HIV infection and PTB and newborn stunting differed according to the type of dietary supplementation received during pregnancy. Maternal HIV was not associated with PTB if the mothers received LNS or IFA; or newborn stunting if the mothers received LNS.

The main weakness of our study was that there was significant loss to follow up whereby only 70% of participants were eligible for inclusion in the analysis. Additionally, there were differences in some of the baseline characteristics between those who were included and those excluded in the analysis. The differences in socioeconomic status score, age and BMI were unlikely to have affected our results because socioeconomic status score was not associated with birth weight in this population and the differences in age and BMI were probably too small to be clinically meaningful. However, considering that the proportions of primigravidity and anaemia were much higher among the excluded women and that they are both associated with foetal growth faltering, we may have underestimated the impact of HIV on birth size. However, after controlling for characteristics that differed between the included and excluded participants, our adjusted results did not change for the most part. Further, the rate of attrition did not differ by HIV infection and intention-to-treat-analysis supported the findings that HIV was associated with FGR.

A limitation of these analyses was that the main iLiNS-DYAD-Malawi trial was not powered for the stratified analyses and the study may have lacked sufficient power to detect differences in the incidence of relatively uncommon dichotomous outcomes such as PTB and LBW. Therefore, failure to observe a significant association between HIV and PTB or LBW among women who received IFA or MMN (for LBW only) needs to be interpreted with caution.

Even though we could determine ART status in less than half of the women, we were able to show that maternal ART status was evenly distributed in the nutrient supplement groups. Analysis restricted to ART naïve HIV positive women showed weaker associations between HIV and birth size but a stronger association with PTB. This concurs with observations from the Tanzania study where ART use was associated with higher rates of LBW and SGA but lower or similar rates of PTB [19]

It was not surprising to observe a significantly lower rate of GWG among the HIV infected women. Pregnancy itself is associated with increased energy requirements [21]. In addition, the increased energy requirements [22] and reduced dietary intake, probably due mainly to reduced appetite [23], that are associated with HIV infection may lead to slower weight gain especially in resource-limited settings where the food intake may not be sufficient to meet the daily needs. Provision of LNS was not associated with improvement in the rate of GWG among HIV infected or HIV uninfected women. This is similar to findings among healthy women in Ghana [24] but not among healthy women in Bangladesh where antenatal LNS improved the rate of GWG, though only among multiparous women ≥ 25 years [25].

Similar to findings from a recent meta-analysis, our study showed that maternal HIV infection was associated with the occurrence of PTB and LBW [3]. Like in our study, women included in the meta-analysis had variable ART use and treatment with ARV drugs did not appear to change the relationship between maternal HIV infection and PTB or LBW [3]. We identified one previous study that compared newborn stunting and underweight between HIV exposed and HIV unexposed babies in Tanzania [26]. The overall rate of stunting was higher in Tanzania than in our Malawi study population. In contrast, the overall rate of underweight was almost twice as high in Malawi. These differences may be due to differences in background characteristics between the two study populations. Similar to this study, maternal HIV was associated with newborn stunting but not with newborn underweight. The failure to observe an association with newborn underweight in the Malawi and Tanzania studies may have been due to insufficient study power to detect a difference considering that the rates of underweight were very low in these countries.

Maternal HIV infection was not associated with newborn head circumference in our study. This is similar to reported observations in Tanzania [26]. However, earlier studies conducted in the pre-ART era found that maternal HIV infection was associated with head circumference [20]. Maternal ART may protect against the adverse effects of HIV on foetal head growth by preventing the progression of HIV disease. Even though maternal ART use was not associated with newborn head circumference in this study, it is possible that widespread availability of ART allows HIV infected women to access treatment before progression of HIV disease to advanced stages which have been associated with adverse outcomes earlier [27,28].

The lack of association between maternal HIV and pregnancy outcomes was not limited to women who received LNS but was also observed for some outcomes among those who received either MMN or IFA. Nevertheless, there is a consistent pattern whereby values of birth size or the risk of adverse birth outcomes among HIV infected women who received LNS are closer to those of HIV negative women irrespective of type of supplement received than they are to those of HIV positive women who received either MMN or IFA. This observation suggests that LNS may be protective against the negative effects of maternal HIV infection on foetal growth. HIV infection is associated with lower maternal weight [29], anaemia [30], oxidative stress [9,31] and micronutrient deficiencies [32,33]. The first three have been shown to be associated with poor foetal growth [11,34,35], and presumed reversal of micronutrient deficiencies by MMN supplementation during pregnancy has resulted in larger birth size in multiple clinical trials [36]. LNS may prevent HIV associated adverse birth outcomes by reducing the risk of one or more of these four factors.

With regard to maternal weight, women with inadequate GWG according to IOM guidelines were more likely to have SGA or LBW babies in the USA [37] and Taiwan [38]. In subgroup analysis of this study, prevalence of inadequate GWG among HIV infected women was similar between those who received LNS and those who received IFA. This therefore makes it unlikely that LNS improved foetal growth among infected women through increases in GWG.

Antenatal iron use is associated with reduced risk of maternal anaemia [39]. Receiving LNS (with 20 mg iron) in the iLiNS-DYAD-Malawi trial was associated with a higher risk of maternal anaemia at 36 gestation weeks, compared to IFA with 60 mg of iron [40]. This therefore indicates that LNS could not have prevented FGR by reducing the risk of maternal anaemia in this study.

It is unlikely that improvement in maternal micronutrient status due to LNS explains our findings because the MMN group received the same daily dose of 18 micronutrients as the LNS group, yet the risk of stunting or SGA was lower in the LNS group than in the MMN group. This suggests that other factors such as the macrominerals or essential fatty acids that were present in LNS and not in the MMN may have been responsible for preventing FGR in HIV infected women.

There is insufficient evidence to conclusively demonstrate that antenatal supplementation with macrominerals improves foetal growth. A meta-analysis that reviewed 15 randomised controlled trials showed that antenatal supplementation with calcium was associated with a modest increase in birth weight but no impact on intrauterine growth restriction was observed [41]. There is little research on the association between phosphorus or potassium supplementation and foetal growth. On the other hand, a Cochrane review showed no impact of antenatal magnesium on the occurrence of SGA [42]. More research on the role of macrominerals with regard to foetal growth is needed to increase our understanding of whether prenatal supplementation with such nutrients is beneficial.

While the LNS differed in protein and energy content compared to the IFA, the difference was probably not large enough to affect foetal growth in a population that likely does not meet the estimated average requirement of nutrients as observed in a study that was conducted close to our study area [43]. The level of protein (2.6 g) in the LNS was much less than the estimated average requirement of protein in early and late gestation (1.2g/kg and 1.54g /kg respectively) [44] and the extra 118 kcal of energy was unlikely to meet the dietary deficits of HIV pregnant women [45]. A recent observational study in Australia showed that maternal intake of alpha linolenic acid was lowest in the group that had SGA babies compared to those who had appropriate- or large-for-gestational-age babies [46] suggesting improved foetal growth in women who had a higher intake of alpha linolenic acid. In our study, however, improved foetal growth was observed only in HIV infected women who received LNS and not in the uninfected women who received LNS. It is probable that in our study population essential fatty acids may have prevented FGR in HIV infected women through other mechanisms as explained below.

It is possible that the LNS reduced the occurrence of HIV associated adverse birth outcomes through the effects of alpha linolenic acid. Oxidative stress is associated with FGR through oxidative damage and inflammation to placental tissue which leads to placental insufficiency [11]. An increase in markers of oxidative stress has been reported in individuals with HIV infection when compared to healthy subjects [9]. In the main iLiNS-DYAD-M trial, LNS provision increased the plasma levels of alpha linolenic acid, a precursor of docosahexanoic acid and eicosapentanoic acid. These long chain n-3 polyunsaturated fatty acids are associated with antioxidant and anti-inflammatory activities [47,48]. Thus, LNS may have decreased oxidative stress and inflammation, thereby reducing FGR among HIV positive women.

These data indicate the potential role antenatal LNS may have in reducing HIV-associated adverse birth outcomes in the era of widespread use of ART. In Tanzania, antenatal supplementation with multivitamins (B1, B2, B6, niacin, B12, C, E and folic acid) reduced the risk of LBW, PTB and SGA among women who had no access to ART [49]. Our study also demonstrates that HIV infected pregnant women who may not be eligible for nutritional supplements according to guidelines of some countries, e.g. do not qualify as undernourished based on MUAC, may have improved birth outcomes if they receive antenatal small-quantity LNS. Only 4.5% of the HIV infected women in our study would have qualified for nutritional supplements according to the current guidelines for nutritional support in Malawi which recommend nutritional supplements only to pregnant women with moderate undernutrition (MUAC: 190–219 mm) or severe undernutrition (MUAC<190 mm) [50].

## Conclusions

Further research to investigate the impact of LNS on various aspects of foetal growth in HIV infected women is warranted.

## Supporting information

S1 Table Baseline characteristics of included and excluded participants. (DOCX)
