# Supplementary material for: Lipid based nutrient supplements during pregnancy may improve foetal growth in HIV infected women – A cohort study
Source: PLoS One. 2019 May 2;14(5):e0215760. doi: 10.1371/journal.pone.0215760 (PMC6497372; doi:10.1371/journal.pone.0215760)
Supplement: S1 Table — (DOCX) [file pone.0215760.s001.docx]

**S1 Table. Baseline characteristics of included and excluded participants**

| **Characteristic** | **Included** | **Excluded** | **P-value^a^** |
| --- | --- | --- | --- |
| Number of participants | 967 | 424 |  |
| Mean (SD) height (cm) | 156.2 (5.7) | 155.6 (5.6) | 0.067 |
| Mean (SD) BMI (kg/m²) | 22.0 (2.7) | 22.5 (3.0) | **0.015** |
| Mean (SD) MUAC (cm) | 26.3 (2.5) | 26.5 (2.9) | 0.193 |
| Mean (SD) maternal age, years | 25.4 (6.1) | 23.9 (6.1) | **<0.001** |
| Mean (SD) maternal educational achievement (completed years at school) | 4.0 (3.4) | 4.0 (3.5) | 1.000 |
| Mean (SD) proxy for SES | -0.05 (0.95) | 0.15 (1.13) | **0.002** |
| Anaemia (Hb < 100 g/l), % | 19.0 | 24.4 | **0.023** |
| Primigravid, % | 17.5 | 31.8 | **<0.001** |
| Low BMI (< 18.5 kg/m²), % | 5.4 | 5.2 | 0.888 |
| P. falciparum infection (RDT), % | 22.2 | 25.6 | 0.163 |
| HIV, % | 14.0 | 13.1 | 0.694 |

BMI, body-mass-index; MUAC, mid-upper arm circumference; SES, socioeconomic status.

^a^ P-values were obtained from t-test (comparison of means) or Chi square test (comparison of proportions).
